# Comparative Bioactivities and Fatty Acid Composition of *Pinus koraiensis* Leaf Oils Obtained Using Different Extraction Methods

**DOI:** 10.3390/life16010049

**Published:** 2025-12-27

**Authors:** Jung-Eun Kim, Kyung Tae Jang, Leeseon An, Min-Ho Lee, Hyo-Jeong Lee

**Affiliations:** 1 Department of Science in Korean Medicine, Graduate School, College of Korean Medicine, Kyung Hee University, 26, Kyungheedae-ro, Dondaemun-gu, Seoul 02447, Republic of Korea; kimjulie4717@khu.ac.kr (J.-E.K.); iseony77@khu.ac.kr (L.A.); 2 Department of Food and Cooking Science, Sunchon National University, 255 Jungang-ro, Suncheon-si 57922, Jeollanam-do, Republic of Korea; jangkt@scnu.ac.kr; 3 Department of Food Science and Biotechnology, Eulji University, 553, Sanseong-daero, Sujeong-gu, Seongnam-si 13135, Gyeonggi-do, Republic of Korea; minho@eulji.ac.kr; 4 Department of Cancer Preventive Material Development, Graduate School, College of Korean Medicine, Kyung Hee University, 26, Kyungheedae-ro, Dondaemun-gu, Seoul 02447, Republic of Korea

**Keywords:** *Pinus koraiensis*, essential oil, supercritical CO_2_ extract

## Abstract

*Pinus koraiensis* leaves are known for various bioactivities, including anti-cancer, anti-obesity, anti-diabetic, and anti-hyperlipidemic effects. This study aimed to compare the essential oil from *P. koraiensis* leaves (EPO) and the supercritical-CO_2_-extracted oil (SPO) for physicochemical traits, antibacterial and anticancer activities, and anti-inflammatory/antioxidant effects, and profiled fatty acids by means of GC-MS. SPO showed stronger antimicrobial activity than EPO against *Streptococcus mutans*, whereas EPO was more active against *Candida albicans*. In HaCaT keratinocytes and THP-1 monocytic cell line, SPO more effectively suppressed LPS-induced ROS and attenuated TNF-α and IL-6 upregulation. Across a panel of human cancer cell lines, SPO exerted greater cytotoxicity, particularly in non–small cell lung, prostate, and colon cancers. GC–MS revealed greater compositional diversity in SPO (16 fatty acids, 10 unique), while linolelaidic acid was detected only in EPO; pentadecenoic acid was abundant in all oils. Collectively, SPO demonstrates broader bioactivity and richer fatty-acid diversity than EPO, supporting its potential as a functional food or medicinal ingredient.

## 1. Introduction

Recently, the importance of medicinal plants has regained global attention as approaches to health promotion and disease prevention continue to diversify. Plants have served as one of the oldest therapeutic resources in human history and can exhibit broad biological activities, such as antioxidant, anti-inflammatory, immunomodulatory, and antimicrobial effects, through a wide range of bioactive metabolites [1,2,3]. In particular, natural products derived from plants can be utilized in diverse forms, including crude extracts, fractions, and essential oils. Owing to the potential synergistic effects of multi-component compositions and a possibly lower toxicity profile, plant-based resources are increasingly regarded as key candidates for the development of functional foods and natural product-based therapeutics.

In contrast, although chemical or synthetic medicines have played crucial roles in disease treatment, their limitations have been continuously highlighted, including adverse effects associated with long-term use, drug resistance, and multi-organ burden. Against this background, interest in herbal, organic, and nature-derived alternatives has expanded worldwide as complementary or supportive strategies that aim to maintain therapeutic efficacy while improving safety and sustainability. Essential oils, in particular, contain bioactive constituents such as monoterpenes, sesquiterpenes, and phenolic compounds, which are potentially linked to regulating cellular signaling and mitigating oxidative stress, thereby increasing their academic and industrial value as candidates for natural antioxidant and anti-inflammatory agents [4,5].

Within this context, *Pinus. koraiensis* (Korean pine) is an evergreen conifer native to Northeast Asia and holds substantial ecological and economic value as well as promising potential as a medicinal resource. *P. koraiensis* leaf-derived materials have been reported to exert various biological effects, including antioxidant [6], anticancer [7,8], anti-obesity [9,10,11], anti-diabetic [12], anti-hyperlipidemic [10,11,13,14], anti-fatigue [15,16], anti-wrinkle [17,18], and antimicrobial activities [6,19]. Beyond its medicinal potential, this species contributes to forest ecosystem stability and regional biodiversity, highlighting the importance of its cultivation and conservation.

However, most bioactivity investigations of *P. koraiensis* leaves have primarily focused on ethanol extracts or ethyl acetate fractions, while comparative evidence on extraction-dependent differences in leaf oils remains limited. Importantly, steam distillation is expected to enrich predominantly volatile constituents and may alter thermolabile compounds due to heat exposure, whereas supercritical CO_2_ extraction may recover a broader spectrum of lipophilic components, including less volatile and heat-sensitive molecules. These extraction-driven compositional differences provide a clear biological and mechanistic rationale for anticipating distinct antioxidant/anti-inflammatory activities and fatty acid profiles between the two oils. To address this gap, the present study evaluated the bioactivities and fatty acid profiles of two *P. koraiensis* leaf oils obtained using different extraction methods, an essential oil produced by means of steam distillation (EPO) and an oil obtained via supercritical CO_2_ extraction (SPO). We aimed to clarify extraction-dependent functional differences and provide mechanistic evidence supporting the potential of *P. koraiensis* leaf oils as functional, plant-based bioresources.

## 2. Materials and Methods

### 2.1. Preparation of EPO and SPO

Dried *Pinus koraiensis* leaves from >30-year-old trees harvested in Hongcheon-gun, Gangwon-do, Republic of Korea, were purchased from Beaksongyounglim (Chuncheon-si, Republic of Korea) for EPO and SPO. EPO was prepared by hydrodistillation. Dried, pulverized *P. koraiensis* leaves were immersed in distilled water and steam-distilled in a condenser-equipped apparatus (Hanil Labtech, Seoul, Republic of Korea) at 90 °C for 3–4 h. The distillate was allowed to stand for 20 min to permit phase separation, after which the essential oil layer was collected and purified by microfiltration. The yield of EPO was approximately 1% (*w/w*). SPO was extracted from dried and pulverized *P. koraiensis* leaves using supercritical carbon dioxide (SC-CO_2_) extraction. Approximately 600 g of pulverized leaves were placed in a 2000 mL extractor. Liquid CO_2_ pre-cooled to 0 °C was compressed and introduced into the extractor maintained at the desired temperature using a thermostat. The pressure was regulated using back-pressure regulators, and the CO_2_ flow rate was maintained at 0.19–0.21 g/s. The extracted oil was collected from the pressurized separator, weighed right after collection, and then stored at 253 K prior to component analysis. Extraction was conducted for 4 h under various conditions: temperatures of 40, 60, and 80 °C, pressures of 10, 20, and 30 MPa. The yield of SPO under optimal conditions was approximately 3% (*w/w*).

### 2.2. Disk Diffusion Assay

The antimicrobial activity of EPO and SPO extract against *Streptococcus mutans* and *Candida albicans* was evaluated using the agar disc diffusion method. A suspension of *C. albicans* (1.28 × 10^8^ CFU/mL) was evenly spread onto yeast malt (YM) agar plates, while *S. mutans* (5.1 × 10^8^ CFU/mL) was inoculated onto Lysogeny Broth (LB) agar plates. Sterile filter-paper disks (10 mm in diameter) were placed on the surface of each inoculated plate and subsequently impregnated with 60 μL of EPO or SPO extract at a concentration of 100 mg/mL. As a control, 60 μL of dimethyl sulfoxide (DMSO) was used. The plates were incubated for at 37 °C for 72 h under anaerobic conditions and the zones of inhibition were measured to assess antimicrobial activity as previously described [20].

### 2.3. Cell Cultures

HaCaT and A375P cells (Korean Cell Line Bank, Seoul, Republic of Korea) were cultured in DMEM medium containing 10% fetal bovine serum (Cat: S101-07, WELGENE, Daegu, Republic of Korea) and 1% antibiotics (Cat: LS203-01, WELGENE, Daegu, Republic of Korea). THP-1, SW620. HCT116, A549, H460, PC-3, DU145, and MDA-MB231 cells (Korean Cell Line Bank, Seoul, Republic of Korea) were cultured in RPMI-1640 (Cat: LM 011-01, WELGENE, Daegu, Republic of Korea) medium with 10% fetal bovine serum and 1% antibiotics. All cells were cultured in a humidified atmosphere with 5% CO_2_ at 37 °C.

### 2.4. Cell Viability Assay

Cells (1 × 10^4^ cells/well) were seeded in 96-well plates and incubated for 24 h. Subsequently. The cells were treated with indicated concentrations of EPO and SPO (0, 3.1, 6.3, 12.5, 25, 50, 100, and 200 µg/mL) and incubated for 24 h. Cell viability was evaluated using a CELLOMAX^TM^ viability kit (Cat: CM-VA2500, Precaregene, Daejeon, Republic of Korea). The absorbance was measured at 450 nm using a microplate reader (Sunrise RC, Tecan, Mannedorf, Switzerland) and determined as previously described [20,21]. Data are expressed as the mean ± SD from triplicate experiments (*n* = 3). One-way ANOVA with Tukey’s test was used. * *p* < 0.05, ** *p* < 0.01, *** *p* < 0.001 vs. control.

### 2.5. Measurement of ROS

ROS formation was evaluated using the probe 2′7′- dichlorofluorescein diacetate (DCF-DA) (Abcam, Waltham, MA, USA). HaCaT and THP-1 (5 × 10^4^) cells were seeded in an 8-well plate and incubated for 24 h. The cells were treated with SPO or EPO (25 µg/mL) with LPS (1 µg/mL) in phenol-free medium containing 10% bovine serum medium for 24 h. Additionally, Tert-butyl hydroperoxide (TBHP) was treated for 4 h as a positive control for ROS production. After incubation, the DCF-DA assay was performed as previously described [20]. Data are expressed as the mean ± SD from triplicate experiments (*n* = 3). One-way ANOVA with Tukey’s test was used (*p* < 0.05); different letters indicate significance.

### 2.6. Western Blot Analysis

The Western blot analysis was performed as previously described [20,22]. Cells were lysed with RIPA buffer, and an equal amount of protein was separated on 15% SDS-polyacrylamide gels and transferred onto a nitrocellulose membrane (Cat: 10600001, Amersham Pharmacia, Piscataway, NJ, USA). Membranes were blocked with 5% skim milk for 1 h and incubated with primary antibodies (Table 1) overnight at 4 °C, followed by the secondary antibodies for 2 h at room temperature. Protein bands were visualized using an enhanced chemiluminescence (ECL) system (GE Healthcare Life Sciences, Marlborough, MA, USA), and Image J software (version 1.53, National Institute of Health, MD, USA) was used to quantify each protein band. Data are expressed as the mean ± SD. One-way ANOVA with Tukey’s test was used (*p* < 0.05); different letters indicate significance.

### 2.7. GC Analysis

The GC analysis was performed using a SACTM-5 Fused Silica Capillary Column (30 m × 0.25 mm × 0.25 µm, Supelco, Bellefonte, PA, USA), and a flame ionization detector. The temperature of the detector was set to 300 °C, and the oven temperature was maintained at 285 °C for 20 min. Helium gas was used as carrier gas, the flow rate was 1.0 mL/min, and the final injection amount was 1 μL. Lipid samples were injected, followed by a commercial standard FAME mix, (Cat: CRM 47885, Sigma-Aldrich, St. Louis, MO, USA) that was for identification and quantification of FAMs. Compounds were identified by comparing retention times and mass spectra with those in the NIST library. Quantification was performed by peak area normalization and expressed as relative percentages of total detected compounds.

### 2.8. GC-MS Analysis

GC–MS analysis of EPO and SPO was performed at the National Instrumentation Center for Environmental Management (NICEM), Seoul National University (Seoul, Republic of Korea). A Thermo Scientific Trace 1310 gas chromatograph coupled to an ISQ LT mass spectrometer was used, equipped with a DB-5MS capillary column (Agilent J&W Scientific, Santa Clara, CA, USA, 60 m × 0.25 mm × 0.25 μm). Total ion chromatograms were acquired over an analysis time of approximately 100 min. Mass spectra were recorded in electron-impact (EI) full-scan mode over an m/z range of 35–550. FAME standard mix (Cat: CRM 47885, Sigma-Aldrich, St. Louis, MO, USA) and reference spectra in the NIST mass spectral library were used as references for the identification of fatty acid methyl esters (FAMEs), and quantification was based on peak-area normalization and expressed as relative percentages of total detected compounds.

### 2.9. Integrative Target Prediction and Pathway Analysis

Genes associated with antioxidant, anti-inflammatory, anticancer, and antimicrobial functions were retrieved from GeneShot, and the intersection of these four categories was defined as the core multifunctional gene set. The chemical constituents of *P. koraiensis* (SPO and EPO) were obtained from the experimentally determined fatty-acid composition in our dataset. Each identified compound was subjected to target prediction using SwissTargetPrediction (Homo sapiens). To ensure reliability, only targets with a probability score ≥ 0.1 were retained for downstream analyses. In parallel, literature-curated compound–gene associations were collected from the Comparative Toxicogenomics Database (CTD). SwissTargetPrediction-derived targets and CTD-derived targets were processed independently. Overlapping genes between the two datasets were subsequently identified and used for integrated analyses. Protein–protein interaction (PPI) networks were constructed using STRING with a confidence threshold of > 0.4. Functional enrichment analyses were performed using Enrichr to obtain pathway-level signatures across KEGG and Reactome libraries. In addition, KEGG and Reactome over-representation analyses were independently conducted for the SwissTargetPrediction gene set, the CTD gene set, and their intersection using false-discovery-rate (FDR) adjusted significance thresholds.

### 2.10. RNA Isolation and qRT-PCR

Total RNA was extracted from HaCaT, THP-1, SW620, and A375P cells. HaCaT (2 × 10^5^ cells/well) and THP-1 cells (5 × 10^5^ cells/well) were seeded into 6-well plates and incubated for 24 h, followed by treatment with SPO or EPO (25 µg/mL) in the presence of LPS (1 µg/mL). SW620 and A375P cells (2 × 10^5^ cells/well) were seeded under the same conditions and treated with SPO or EPO (100 µg/mL) for 24 h. Total RNA was isolated using QIAzol Lysis Reagent (Cat: 79306, QIAGEN, Germantown, MD, USA) according to the manufacturer’s instructions. cDNA was synthesized from 1 μg of total RNA using the High-Capacity cDNA Reverse Transcription Kit (Cat: 3122, Bioneer, Seoul, Republic of Korea). Quantitative real-time PCR (qRT-PCR) was performed with the SYBR Green RT-PCR Kit (Cat: 6252, Bioneer, Seoul, Republic of Korea) on a Thermal Cycler Dice Real-Time System III (Takara Bio, Shiga, Japan). qRT-PCR was performed at 95 °C for 10 min, then 40 cycles of 95 °C for 15 s and 60 °C for 60 s. Specificity was confirmed by melt curve analysis, and efficiency (90–110%) by standard curves. Gene-specific primers were used to amplify iNOS (forward 5′-GCT CTA CAC CTC CAA TGT GAC C-3′; reverse 5′-CTG CCG AGA TTT GAG CCT CAT G-3′) and β-actin (forward 5′-AAG AGA GGC ATC CTC ACC CT-3′; reverse 5′-ATC TCT TGC TCG AAG TCC AG-3′). All experiments were performed in triplicate (*n* = 3). Data are expressed as the mean ± SD. One-way ANOVA with Tukey’s test was used (*p* < 0.05); different letters indicate significance.

### 2.11. Statistical Analysis 

The data presented in this study are expressed as the mean ± standard deviation (S.D.) and were obtained from three replicates for each experiment. Analysis of variance was used to assess the significance of differences between groups. Statistical significance was set at *p* < 0.05. Significance was evaluated using Sigma Plot software (version 14: Systat Software Inc., San Jose, CA, USA). Data were analyzed by one-way ANOVA, followed by Tukey’s studentized range test using GraphPad Prism software (version 8: GraphPad Software Inc., San Diego, CA, USA).

## 3. Results

### 3.1. Comparison of the Antimicrobial Activities of Pinus koraiensis Essential Oil (EPO) and Supercritical CO_2_ Extract Oil (SPO) Against Streptococcus mutans and Candida albicans

**Figure 1:**
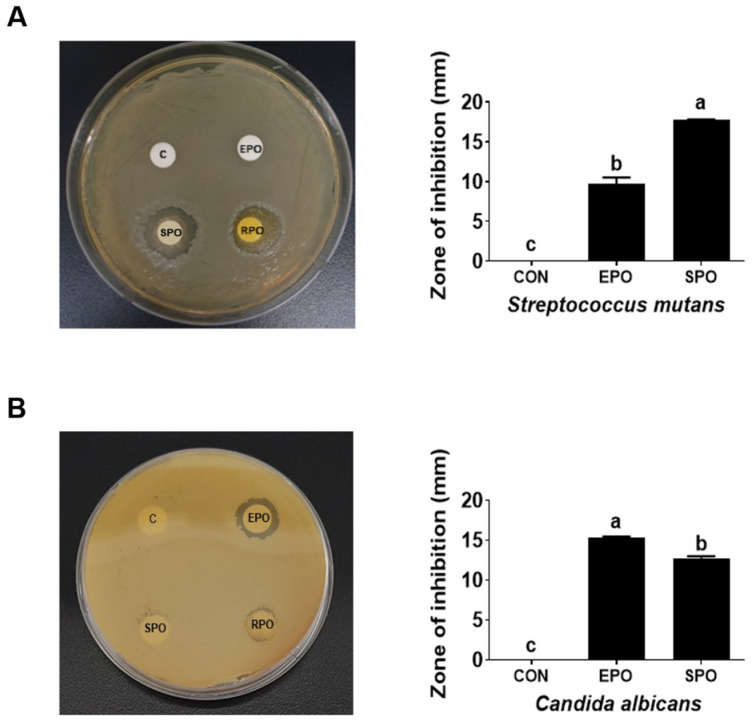
Comparison of the antimicrobial activities of *Pinus koraiensis* essential oil (EPO) and supercritical CO_2_ extract oil (SPO) against (**A**) *Streptococcus mutans* and (**B**) *Candida albicans*. Paper disks loaded with DMSO (CON), EPO, or SPO were tested by agar diffusion. The zone of inhibition is presented as the mean ± SD. Different letters indicate significant differences (*p* < 0.05, one-way ANOVA with Tukey’s test).

The antimicrobial activities of EPO and SPO were evaluated against two clinically relevant microorganisms: *Streptococcus mutans*, a key contributor to tooth decay, and *Candida albicans*, an opportunistic pathogenic yeast implicated in fungal urinary tract infections (UTIs), using the agar diffusion method at a dose of 6 mg/disk. Both EPO and SPO exhibited potent activity against *Streptococcus mutans* and *Candida albicans* (Figure 1A,B and Table 2). EPO produced zones of inhibition of 10 mm and 15 mm against *S. mutans* and *C. albicans*, respectively, while SPO yielded zones of 18 mm and 12 mm against *S. mutans* and *C. albicans,* respectively (Figure 1A,B). Therefore, EPO exhibited greater antimicrobial activity than SPO against *Candida albicans*, whereas SPO was more active than EPO against *Streptococcus mutans*.

### 3.2. Comparison of the Antioxidant Activities of EPO and SPO on LPS-Stimulated HaCaT and THP-1 Cells

**Figure 2:**
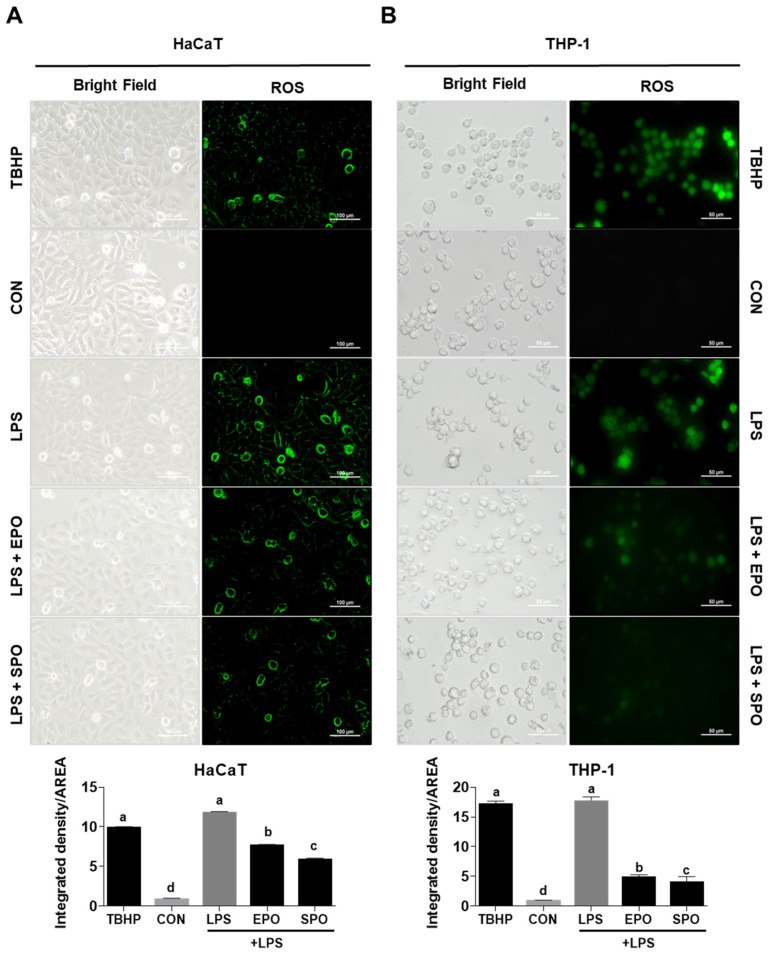
Comparison of the antioxidant activities of *Pinus koraiensis* essential oil (EPO) and supercritical CO_2_ extract oil (SPO) on LPS-stimulated HaCaT and THP-1 cells. (**A**) HaCaT and (**B**) THP-1 cells were treated with LPS, LPS + EPO, or LPS + SPO (25 μg/mL) for 24 h. TBHP (50 μM) was used as a positive control. Intracellular reactive oxygen species (ROS) were detected using the DCFH-DA probe, and fluorescence images were captured by fluorescence microscopy. The bar graphs represent quantitative analysis of fluorescence intensity (integrated density/area). Data are presented as the mean ± SD (*n* = 3). Different letters indicate significant differences (*p* < 0.05, one-way ANOVA).

Bacterial infection generates reactive oxygen species (ROS) both from bacteria and from the host immune system. Bacteria produce ROS as metabolic byproducts and can deploy them to kill competitors [23]. Lipopolysaccharide (LPS), a major outer-membrane component of bacterial cell walls, robustly induces ROS across multiple cell types, particularly immune cells such as macrophages and endothelial cells [24,25]. The ROS generation is central to oxidative stress and inflammatory responses. In our study, LPS increased ROS levels by 12-fold in HaCaT keratinocytes and 10-fold in THP-1 cells compared to untreated controls (*p* < 0.05). Treatment with EPO and SPO reduced ROS production in HaCaT cells by 4-fold and 6-fold, respectively. In THP-1 cells, ROS levels were suppressed by 6-fold with EPO and 7-fold with SPO (Figure 2A,B).

### 3.3. Comparison of the Anti-Inflammatory Effect of EPO and SPO on LPS-Stimulated HaCaT and THP-1 Cells

**Figure 3:**
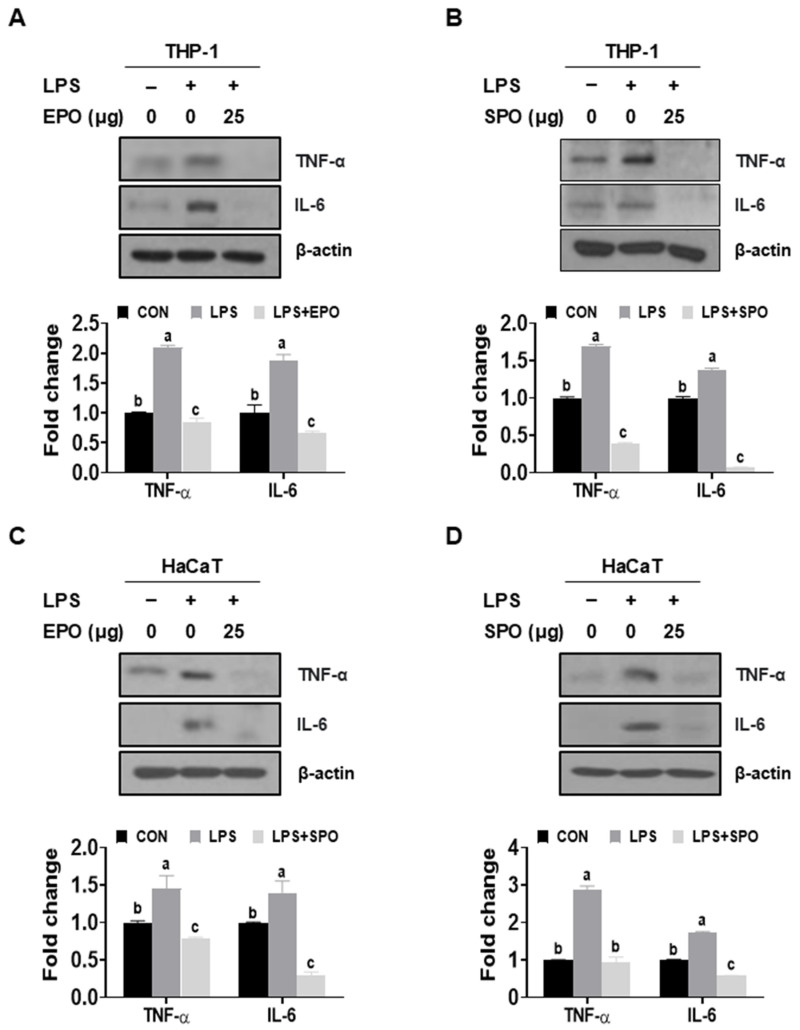
Comparison of the anti-inflammatory effects of *Pinus koraiensis* essential oil (EPO) and supercritical CO_2_ extract oil (SPO) on LPS-stimulated THP-1 and HaCaT cells. (**A**,**B**) THP-1 and (**C**,**D**) HaCaT cells were treated with or without LPS for 24 h. LPS-stimulated cells were co-treated with either (**A**,**C**) EPO or (**B**,**D**) SPO. Protein expression levels of TNF-α, IL-6, and β-actin were analyzed via Western blotting. Data are presented as the mean ± SD. Different letters indicate significant differences (*p* < 0.05, one-way ANOVA).

In THP-1 and HaCaT cells, LPS markedly increased TNF-α and IL-6 protein abundance relative to untreated controls (Figure 3A–D). Co-treatment with EPO or SPO (25 μg/mL) significantly suppressed LPS-induced TNF-α and IL-6 expression in both cell types, reducing levels to near or below baseline (Figure 3A–D). In THP-1 cells, both oils reduced LPS-elevated TNF-α and IL-6 to <~60% of the LPS condition (Figure 3A,B). In HaCaT cells, inhibition was also pronounced, with SPO showing the greatest suppression for both cytokines, approaching or dropping below control levels (Figure 3C,D). Collectively, EPO and SPO attenuated LPS-stimulated proinflammatory signaling in THP-1 and HaCaT cells.

### 3.4. Comparison of Cancer Cell Growth Inhibitory Properties of EPO and SPO on Various Human Cancer Cell Lines

**Figure 4:**
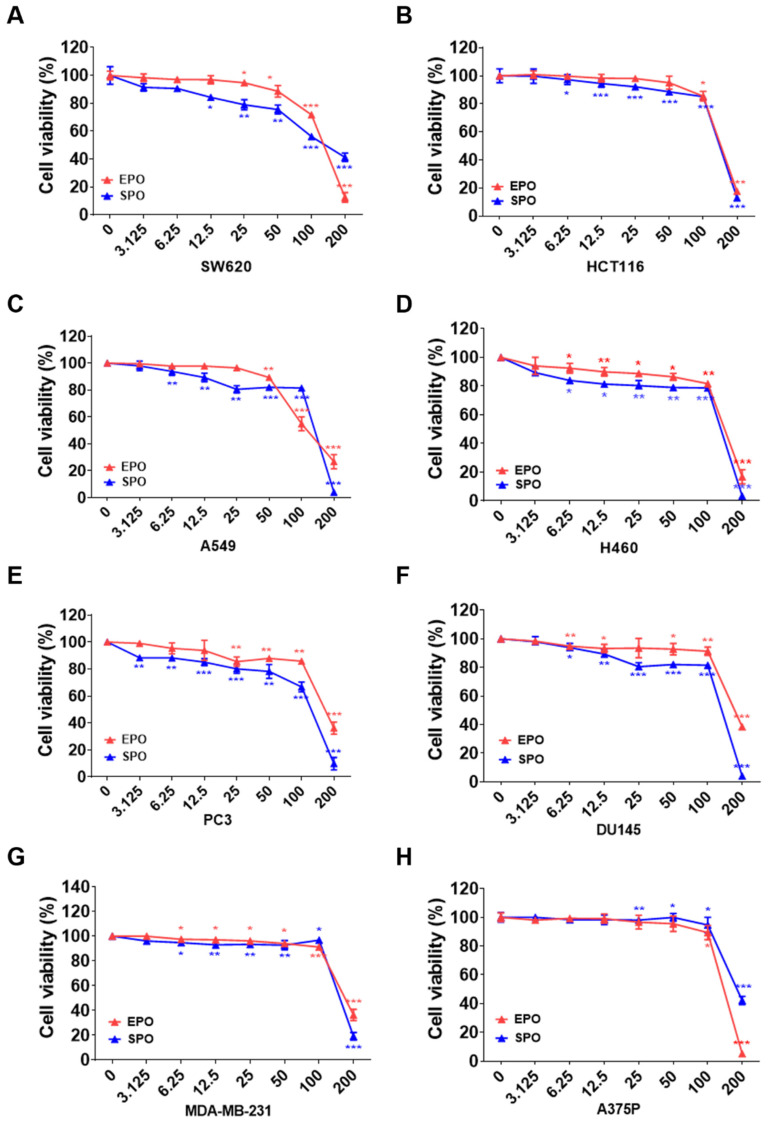
Comparison of the cancer cell growth inhibitory effects of *Pinus koraiensis* essential oil (EPO) and supercritical CO_2_ extract oil (SPO) on various human cancer cell lines. (**A**,**B**) Colon cancer (SW620, HCT116), (**C**,**D**) lung cancer (A549, H460), (**E**,**F**) prostate cancer (PC-3, DU145), (**G**) breast cancer (MDA-MB-231), and (**H**) melanoma (A375P) cells were treated with the indicated concentrations of EPO or SPO for 24 h. Cell viability was measured using the MTS assay, and the results are expressed as the mean ± SD (*n* = 3). * *p* < 0.05, ** *p* < 0.01, and *** *p* < 0.001 versus control groups.

We compared the effects of EPO and SPO on tumor cell viability across eight human cancer cell lines (Figure 4A–H). In all models, colon (SW620, HCTll6), lung (A549, H460), prostate (PC-3, DU145), breast (MDA-MB-231), and melanoma (A375P), both oils reduced cell viability in a clear concentration-dependent manner over 3.125–200 μg/mL. Significant inhibition was already evident from low-mid doses in several lines, and an abrupt drop occurred at ≥100–200, where viability typically fell below 40%. Across most cell types, SPO exhibited greater cytotoxicity than EPO at intermediate concentrations. This trend was most apparent in SW620, H460, PC-3, DU145, and MDA-MB-231, where SPO consistently produced larger decreases in viability than EPO at 12.5–100 μg/mL (Figure 4A–H). To assess cancer-versus-non-cancer selectivity, MTT assays were additionally performed in HaCaT and THP-1 cells, and selectivity indices (SI = IC_50_ in non-cancer cells/IC_50_ in cancer cells) were calculated. Most SI values were >1, indicating that the cancer cell lines were generally more sensitive than the non-cancer models under our conditions; however, SPO showed SI <1 in A375 cells, suggesting that selectivity may be cell type–dependent (Table 3).

### 3.5. Comparison of the Fatty Acid Content and Volatile Terpenoids in the EPO and SPO

**Figure 5:**
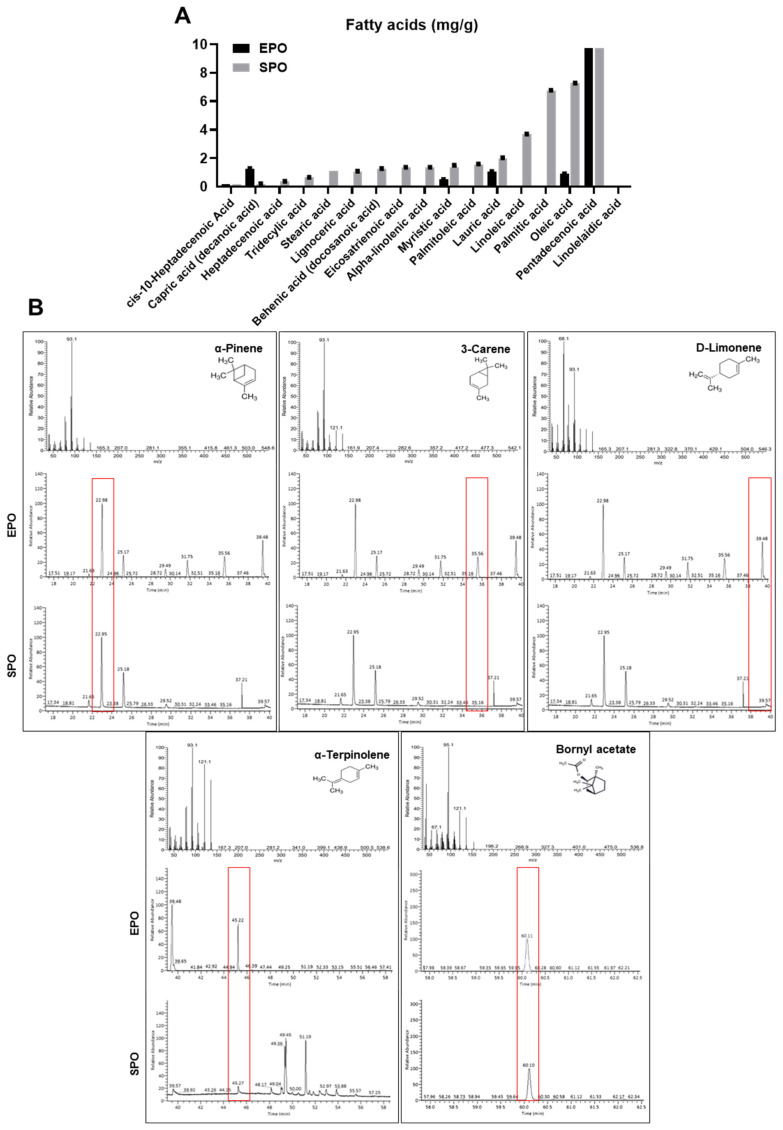
GC and GC–MS identification of fatty acids and major constituents in EPO and SPO. (**A**) Comparison of fatty acid composition between *Pinus koraiensis* essential oil (EPO) and supercritical CO_2_ extract oil (SPO). Fatty acids were analyzed using GC. Data are presented as the mean ± SD (*n* = 3). Black and gray bars represent EPO and SPO, respectively. (**B**) Representative GC–MS chromatograms and EI mass spectra of major constituents identified in EPO and SPO, including α-pinene, 3-carene, D-limonene, α-terpinolene, and bornyl acetate. Red boxes indicate the characteristic retention time windows corresponding to each compound.

Fatty-acid profiling (GC analysis). Quantified values (means ± SD) showed that SPO contained a broader and higher-abundance fatty-acid spectrum than EPO (Table 3 and Figure 4). Across shared analytes, pentadecenoic acid was the predominant species in both oils, and SPO displayed significantly higher levels of pentadecenoic acid and tridecylic acid (*p* < 0.05) (Table 4 and Figure 4). Linolelaidic acid was detected only in EPO, whereas ten additional fatty acids were unique to SPO, yielding 16 species in SPO versus six in EPO (Table 4 and Figure 4). These results indicate greater compositional diversity and enrichment of several medium-chain fatty acids in SPO. GC-MS analysis revealed clear differences in the volatile profiles of the EPO and the SPO (Table 5 and Figure 5). In EPO, five major monoterpenoid constituents were identified, with α-pinene showing the highest relative abundance (23.27 ± 0.53%; RT 22.98 min), followed by D-limonene (12.63 ± 0.24%; RT 39.48 min), 3-carene (10.87 ± 0.23%; RT 35.56 min), α-terpinolene (5.31 ± 0.08%; RT 45.22 min), and bornyl acetate (2.57 ± 0.13%; RT 60.11 min). In contrast, among the same set of marker volatiles, only α-pinene and bornyl acetate were detected in SPO, at 3.32 ± 0.66% (RT 22.95 min) and 5.45 ± 0.46% (RT 60.11 min), respectively, whereas 3-carene, D-limonene, and α-terpinolene were not detected (ND). These data indicate that steam distillation enriched a broader spectrum and higher proportion of monoterpenoid volatiles than supercritical CO_2_ extraction.

### 3.6. Network Pharmacology Analysis Predicts the Target Pathways of EPO and SPO

**Figure 6:**
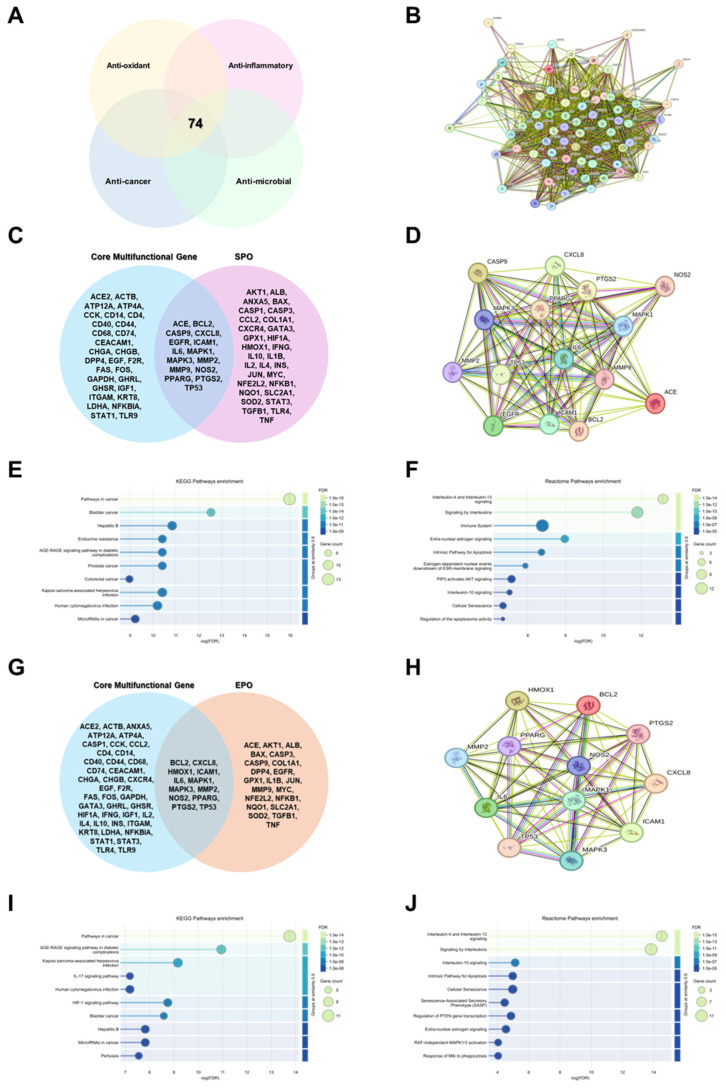
Comparison of the multifunctional gene signatures and pathway networks associated with *Pinus koraiensis* essential oil (EPO) and supercritical CO_2_ extract oil (SPO). (**A**) Overlapping antioxidant, anti-inflammatory, anticancer, and antimicrobial-related gene sets were analyzed to identify 74 core multifunctional genes. (**B**) Protein–protein interaction networks of the 74 core genes were generated using STRING. (**C**,**G**) Core multifunctional genes were compared with SPO or EPO-associated target genes predicted by SwissTargetPrediction and CTD to identify overlapping and unique genes. (**D**,**H**) STRING PPI networks were constructed using the overlapping gene sets obtained from (**C**,**G**) to identify major functional hubs related to inflammation, oxidative stress, and cell survival. (**E**,**I**) KEGG pathway enrichment analyses were performed for SPO and EPO-associated gene sets to determine significantly enriched biological pathways. (**F**,**J**) Reactome pathway enrichment analyses were performed for SPO and EPO-associated gene sets to identify significantly enriched signaling pathways.

To explore convergent mechanisms that may underlie the pleiotropic effects of *Pinus koraiensis* leaf oils, we first integrated targets associated with antioxidant, anti-inflammatory, cancer cell growth inhibitory, and antimicrobial activities reported for EPO and SPO. Using GeneShot with predefined relevance-score thresholds, we retrieved 74 overlapping multi-functional genes. Venn-diagram analysis identified 74 genes shared across all four functional categories (Figure 6A). STRING-based protein-protein interaction (PPI) mapping of these 74 genes suggested a highly interconnected network and highlighted several highly connected nodes, including inflammatory cytokines, matrix metalloproteinases, apoptosis regulators, and key signaling kinases (Figure 6B). In this study, 16 SPO-specific lipid fatty acids and 2 volatile terpenoids (α-Pinene and Bornyl acetate) were analyzed with Swiss Target Prediction and CTD networks to predict putative target genes. Integrating these predicted targets with the 74 overlapping multifunctional genes yielded 15 prediction-derived candidate target genes (ACE, BCL2, CASP9, CXCL8, EGFR, ICAM1, IL-6, MAPK1, MAPK3, MMP2, MMP9, NOS2, PPARG, PTGS2, and TP53) (Figure 6C). The corresponding PPI network indicated MAPK1/3, MMP2/9, PTGS2, CXCL8, and NOS2 as putative highly connected nodes (Figure 6D). Overlapping pathways may be phenotype-specific, but they may also reflect common upstream regulatory mechanisms. KEGG enrichment of these SPO-associated core genes showed significant overrepresentation of cancer- and inflammation-related pathways, including “Pathways in cancer,” “Bladder cancer,” “Hepatitis B,” “Endocrine resistance,” and “AGE–RAGE signaling pathway in diabetic complications,” as well as viral infection pathways such as Kaposi sarcoma-associated herpesvirus and human cytomegalovirus infection (Figure 6E). Reactome analysis similarly pointed to immune and survival signaling, with top terms including “Interleukin-4 and Interleukin-13 signaling,” “Signaling by interleukins,” “Immune system,” “Extra-nuclear estrogen signaling,” “Intrinsic pathway for apoptosis,” “PI3K activates AKT signaling,” and “Cellular senescence” (Figure 6F). A parallel analysis was performed for EPO (EPO-speicific constituents, Capric acid, Lauric acid, Myristic acid, Pentadecanoic acid, Oleic acid, Linolelaidic acid, 3-carene,α-Pinene D-limonene, α-terpinolene, and Bonyl acetate: chosen based on our prior research [14]). Intersection of the 74 multifunctional genes with EPO-specific predicted targets identified 12 prediction-derived candidate core genes (BCL2, CXCL8, HMOX1, ICAM1, IL-6, MAPK1, MAPK3, MMP2, NOS2, PPARG, PTGS2, and TP53) (Figure 6G). The EPO PPI network likewise highligted NOS2, HMOX1, PTGS2, CXCL8, MAPK1/3, MMP2/9, and TNF as densely connected nodes (Figure 6H). KEGG enrichment and Reactome analysis of the EPO-associated core genes showed patterns broadly overlapping with those observed for SPO (Figure 6I). Collectively, these prediction-based analyses are consistent with the possibility that both SPO and EPO may be linked to a shared network related to inflammation, oxidative stress, and cancer-associated signaling, and nominate NOS2 (iNOS) as a common putative candidate target that warrants further experimental validation.

### 3.7. Regulation of iNOS Expression and NO Production by EPO and SPO in Immune and Cancer Cells

**Figure 7:**
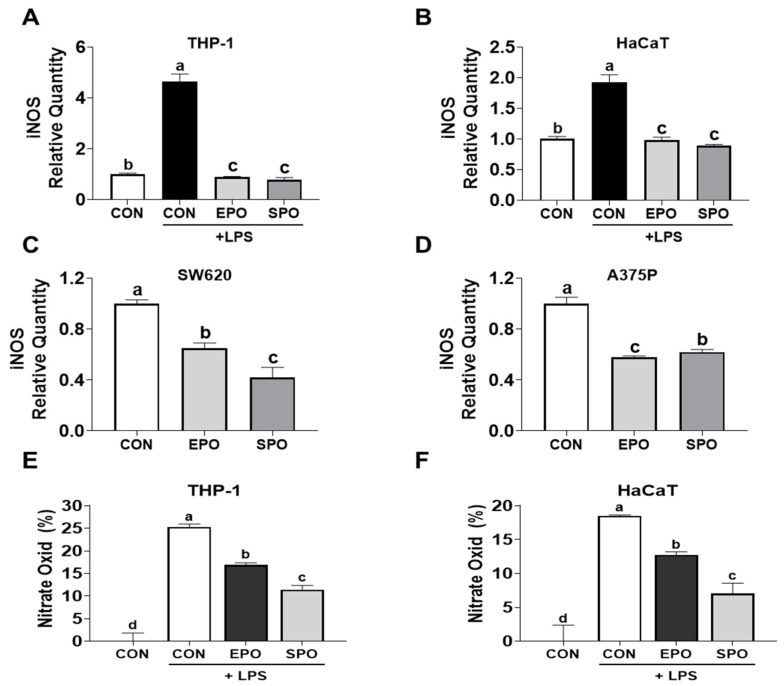
Comparison of the effects of *Pinus koraiensis* essential oil (EPO) and supercritical CO_2_ extract oil (SPO) on iNOS mRNA expression and nitric oxide (NO) production. (**A**) THP-1 and (**B**) HaCaT cells were treated with or without LPS for 24 h, and LPS-stimulated cells were co-treated with EPO or SPO. (**C**) SW620 and (**D**) A375P cells were treated with EPO or SPO for 24 h without LPS stimulation. Relative mRNA expression levels of iNOS were analyzed via qRT-PCR. (**E**,**F**) Nitric oxide levels in LPS-stimulated THP-1 (**E**) and HaCaT (**F**) cells were measured by the Griess assay after co-treatment with EPO or SPO. Data are presented as the mean ± SD (*n* = 3). Different letters indicate significant differences (*p* < 0.05, one-way ANOVA).

In THP-1 cells and HaCaT keratinocytes, lipopolysaccharide (LPS) stimulation markedly increased iNOS mRNA expression compared with unstimulated controls. Treatment with either EPO or SPO significantly attenuated this LPS-induced iNOS upregulation (Figure 7A,B). In human cancer cell lines SW620 and A375P, basal iNOS expression was also reduced by treatment with EPO and SPO (Figure 7C,D). Supporting the transcriptional findings, Griess assay results showed that both EPO and SPO significantly reduced LPS-induced nitric oxide (NO) production in THP-1 and HaCaT cells (Figure 7E,F). Overall, these data support the computational prediction that NOS2 (iNOS) serves as a shared molecular target of both EPO and SPO and demonstrate that both extracts can downregulate iNOS transcription and NO production in inflammatory and tumor cell contexts.

## 4. Discussion

This study provides a head-to-head comparison of *Pinus koraiensis* leaf essential oil (EPO) and supercritical-CO_2_ oil (SPO), integrating antimicrobial, anti-inflammatory, antioxidant, and cancer cell inhibition readouts with fatty-acid profiling. Overall, SPO displayed broader and stronger bioactivity than EPO across assays, whereas EPO showed selective advantages against *Candida albicans* and, in our viability panel, a notable potency in A375P melanoma at high dose. These patterns suggest that extraction modality determines the recovered chemical space, shapes both the spectrum and magnitude of bioactivity.

The superior activity of SPO against *Streptococcus mutans* and its greater suppression of LPS-evoked ROS, TNF-α, and IL-6 in THP-1 and HaCaT cells are consistent with its richer lipid composition (16 fatty acids vs. 6 in EPO, with 10 unique to SPO). Supercritical extraction captures a wider range of neutral and mildly polar lipids, including medium-chain and odd-chain species, that are largely absent from hydrodistilled essential oils dominated by volatile terpenoids [26,27,28]. Medium- and long-chain fatty acids such as capric acid, lauric acid, oleic acid, and linoleic acid enriched in SPO, have been reported antimicrobial effect against *S. mutans,* completely inhibiting biofilm formation and reducing extracellular polysaccharide production [29,30]. Furthermore, these fatty acids have been associated with anti-oxidant [31,32,33,34,35,36,37,38], anti-inflammatory [32,39,40,41,42,43,44,45], and anti-cancer effects [46,47,48,49,50,51,52,53,54,55,56]. These fatty acids may underline SPO’s ability to attenuate LPS-induced oxidative stress and pro-inflammatory cytokine responses, and to reduce cell viability in non-small cell lung, prostate, and colon cancer cell lines.

By contrast, EPO showed greater activity against *C. albicans*. Capric acid, present in both oils but enriched in EPO, has documented anti-microbial activity against *C. albicans*, disrupting or disintegrating fungal membranes, and causing cytoplasmic leakage and cell death [57,58]. Consistent with our findings and prior reports, EPO is enriched for mono-and sesquiterpenes, including limonene, camphene, α-pinene, and borneol [14,59] that intercalate into lipid bilayers and perturb fungal ergosterol-containing membranes [60,61,62,63]. Such constituents may also differentially affect melanoma cells exhibits membranes lipidomes such constituents may also differentially affect melanoma cells, which membrane lipidomes and redox states distinct from those of epithelial cancer cell lines [64,65,66,67,68]. Accordingly, the pronounced effect of EPO in A375P melanoma cells points to terpenoid-centric mechanisms.

Pentadecenoic acid, which is abundant in both oils, has been reported to exhibit anti-inflammatory and antioxidant activities and to support mitochondrial function, thereby reducing oxidative stress in cellular and animal models [69]. It may also exert anticancer effects through multiple mechanisms, including suppression of cancer-stem cell markers and JAK2/STAT3-dependent proliferative signaling, induction of apoptosis, interference with DNA topoisomerase I, and potential epigenetic modulation via HDAC6 inhibition [70].

The network- and pathway-based analysis indicate that EPO and SPO- specific constituents converge on a shared set of multifunctional genes enriched in inflammatory, oxidative-stress, and cancer-associated signaling pathways, with NOS2 emerging as a common putative core target. Consistent with this prediction, both EPO and SPO significantly suppressed LPS-induced iNOS expression in THP-1 and HaCaT cells and reduced basal iNOS levels in SW620 and A375P cancer cells. Consistent with the cancer cell viability data, in which SPO exhibited stronger cytotoxicity than EPO in SW620 cells, whereas EPO more effectively reduced cell viability in A375P cells, the iNOS results likewise showed that EPO suppressed iNOS expression more than SPO in A375P cells, while SPO produced greater inhibition than EPO in SW620 cells. The concordance between the computational prioritization of NOS2 (iNOS2) and its experimentally confirmed downregulation across immune and tumor supports NOS2 (iNOS2) as a key functional effector of these oils. The primary focus of this manuscript is a comparative analysis; therefore, the mechanistic aspects are not fully clarified. Accordingly, further studies will be needed to elucidate the more precise mechanisms of these oils in future work.

In summary, supercritical CO_2_ extraction yields a compositionally diverse *Pinus koraiensis* leaf oil (SPO) with broad antibacterial, anti-inflammatory, antioxidant, and cancer cell inhibitory activities, whereas hydrodistilled essential oil (EPO) confers selective antifungal effects and preferential activity against melanoma. These complementary profiles support extraction method–dependent differences that may guide future development considerations. SPO may be considered for further evaluation as a multi-component source of anti-inflammatory and antimicrobial activity, whereas EPO may warrant follow-up studies focusing on antifungal activity and melanoma cell growth inhibition, pending additional validation.

## Figures and Tables

**Table 1:** Antibodies used in this study.

Antibody	Company	Dilution	Product No.
TNF-α	Cell Signaling	1:1000	3707
IL-6	Cell Signaling	1:1000	12912
β-actin	Sigma-Aldrich	1:20,000	A5316
goat anti-rabbit IgG (HRP)	Abcam	1:5000	ab97051
goat anti-mouse IgG (HRP)	Jackson	1:5000	115-035

Means ± SD Sigma-Aldrich (St Louis, MO, USA); CST, Cell Signaling Technology (Beverly, MA, USA); Abcam (Cambridge, UK); Jackson (West Grove, PA, USA).

**Table 2:** Summary of antibacterial effects against EPO, SPO and RPO using the disk diffusion test.

	Sample	Zone Diameter (mm)		Mean (±SD) mm
***Streptococcus mutans***	DMSO	0	0	0
	EPO	8.97	10.51	9.74 (±1.01)
	SPO	17.78	17.75	17.76 (±0.02)
***Candida albicans***	DMSO	0	0	0
	EPO	15.42	15.23	15.33 (±0.13)
	SPO	12.52	12.97	12.75 (±0.32)

DMSO: Solvent of Sample. Concentration: 100 mg/mL/60 uL per disc.

**Table 3:** IC_50_ values and selectivity indices (SI) of SPO and EPO in cancer and normal cell lines.

Cell Line	SPO IC_50_ (µg/mL)	SPO IC_50_ (µg/mL)	SPO SI		EPO SI	
			HaCaT	THP-1	HaCaT	THP-1
SW620	141.31	136.73	1.19	1.12	1.38	2.00
HCT116	148.73	152.27	1.13	1.06	1.24	1.79
A549	140.69	117.36	1.19	1.12	1.60	2.33
H460	138.09	148.74	1.22	1.14	1.26	1.84
PC-3	129.41	172.12	1.30	1.22	1.09	1.59
DU145	140.69	178.25	1.19	1.12	1.06	1.53
MDA-MB-231	160.21	175.02	1.05	0.99	1.07	1.56
A375P	184.99	146.74	0.91	0.85	1.28	1.86
THP-1	157.93	273.21				
HaCaT	168.07	188.11				

SI = IC_50_ in normal cells (HaCaT or THP-1)/IC_50_ in cancer cells. Values >1 indicate greater sensitivity in cancer cells.

**Table 4:** Fatty acid composition (EPO, SPO mg/g).

Fatty Acid		EPO (mg/g)	SPO (mg/g)
C10:0	Capric acid (decanoic acid)	1.24 ± 0.022	0.20 ± 0.007
C12:0	Lauric acid	1.06 ± 0.001	2.00 ± 0.022
C13:0	Tridecylic acid	ND	0.65 ± 0.005
C14:0	Myristic acid	0.49 ± 0.012	1.37 ± 0.132
C15:0	Pentadecenoic acid	9.80 ± 0.001	9.75 ± 0.001
C16:0	Palmitic acid	ND	6.74 ± 0.028
C16:1	Palmitoleic acid	ND	1.53 ± 0.059
C17:0	Heptadecenoic acid	ND	0.35 ± 0.013
C17:1	cis-10-Heptadecenoic acid	ND	0.16 ± 0.017
C18:0	Stearic acid	ND	1.10 ± 0.006
C18:1n-9, Cis	Oleic acid	0.90 ± 0.011	7.27 ± 0.017
C18:2n-6, Cis	Linoleic acid	ND	3.69 ± 0.006
C18:2n-6, trans	Linolelaidic acid	0.027± 0.001	ND
C18:3n-3	Alpha-linolenic acid (ALA)	ND	1.35 ± 0.011
C22:0	Behenic acid (docosanoic acid)	ND	1.26 ± 0.004
C20:3n-3	Eicosatrienoic acid (ETA)	ND	1.35 ± 0.010
C24:0	Lignoceric acid	ND	1.08 ± 0.004

Means ± SD; ND = not detected.

**Table 5:** GC–MS analysis of major volatile compounds.

	EPO		SPO	
	RT (min)	Area % (Mean ± SD)	RT (min)	Area % (Mean ± SD)
α-Pinene	22.98	23.27 ± 0.53	22.95	3.32 ± 0.66
3-Carene	35.56	10.87 ± 0.23	ND	ND
D-Limonene	39.48	12.63 ± 0.24	ND	ND
α-Terpinolene	45.22	5.31 ± 0.08	ND	ND
Bornyl acetate	60.11	2.57 ± 0.13	60.11	5.45 ± 0.46

Means ± SD; ND = not detected.

## Data Availability

The raw data supporting the conclusions of this article are available from the corresponding author upon reasonable request. The data cannot be publicly released at this stage because they require approval from collaborating authors/institutions, and portions of the dataset are planned for use in ongoing follow-up studies.

## References

[1] Shahbazi R., Sharifzad F., Bagheri R., Alsadi N., Yasavoli-Sharahi H., Matar C. (2021). Anti-inflammatory and immunomodulatory properties of fermented plant foods. Nutrients.

[2] Gangwar V., Garg A., Lomore K., Korla K., Bhat S.S., Rao R.P., Rafiq M., Kumawath R., Uddagiri B.V., Kareenhalli V.V. (2021). Immunomodulatory effects of a concoction of natural bioactive compounds—Mechanistic insights. Biomedicines.

[3] Merecz-Sadowska A., Sadowski A., Zielińska-Bliźniewska H., Zajdel K., Zajdel R. (2025). Network Pharmacology as a Tool to Investigate the Antioxidant and Anti-Inflammatory Potential of Plant Secondary Metabolites—A Review and Perspectives. Int. J. Mol. Sci..

[4] Noriega P. (2020). Terpenes in essential oils: Bioactivity and applications. Terpenes and Terpenoids—Recent Advances.

[5] Valdivieso-Ugarte M., Gomez-Llorente C., Plaza-Díaz J., Gil Á. (2019). Antimicrobial, antioxidant, and immunomodulatory properties of essential oils: A systematic review. Nutrients.

[6] Kim J.-E., Kim W.-Y., Kim J.-W., Park H.-S., Lee S.-H., Lee S.-Y., Kim M.-J., Kim A., Park S.-N. (2010). Antibacterial, antioxidative activity and component analysis of *Pinus koraiensis* leaf extracts. J. Soc. Cosmet. Sci. Korea.

[7] Cho S.-M., Lee E.-O., Kim S.-H., Lee H.-J. (2014). Essential oil of *Pinus koraiensis* inhibits cell proliferation and migration via inhibition of p21-activated kinase 1 pathway in HCT116 colorectal cancer cells. BMC Complement. Altern. Med..

[8] Lee T.K., Roh H.S., Yu J.S., Baek J., Lee S., Ra M., Kim S.Y., Baek K.H., Kim K.H. (2017). Pinecone of *Pinus koraiensis* inducing apoptosis in human lung cancer cells by activating caspase-3 and its chemical constituents. Chem. Biodivers..

[9] Kim E.A., Yang J.-H., Byeon E.-H., Kim W., Kang D., Han J., Hong S.-G., Kim D.-R., Park S.-J., Huh J.-W. (2021). Anti-obesity effect of pine needle extract on high-fat diet-induced obese mice. Plants.

[10] Lee M.-H., Park S., Xu Y., Kim J.-E., Han H., Lee J.-H., Paik J.K., Lee H.-J. (2022). Ethanol extract of *Pinus koraiensis* leaves mitigates high fructose-induced hepatic triglyceride accumulation and hypertriglyceridemia. Appl. Sci..

[11] Park S.-y., Park T.g., Choi K., Kim K.J., Kim J.Y. (2024). The Impact of *Pinus koraiensis* Leaf Extract Consumption on Postprandial ApoB100 and Lipid Metabolism: A Randomized, Double-Blind, Placebo-Controlled Trial in Healthy Participants Subjected to an Oral High-Fat Challenge. Nutrients.

[12] Joo H.-E., Lee H.-J., Sohn E.J., Lee M.-H., Ko H.-S., Jeong S.-J., Lee H.-J., Kim S.-H. (2013). Anti-diabetic potential of the essential oil of *Pinus koraiensis* leaves toward streptozotocin-treated mice and HIT-T15 pancreatic β cells. Biosci. Biotechnol. Biochem..

[13] Kim S., Lee H., Jeong S., Lee E., Lee M. (2011). Antihyperlipidermic and antidiabetic effects of *Pinus koraiensis* leaf oil. Planta Medica.

[14] Kim J.H., Lee H.J., Jeong S.J., Lee M.H., Kim S.H. (2012). Essential oil of *Pinus koraiensis* leaves exerts antihyperlipidemic effects via up-regulation of low-density lipoprotein receptor and inhibition of acyl-coenzyme A: Cholesterol acyltransferase. Phytother. Res..

[15] Lee S.M., Kim Y.H., Kim Y.R., Lee B.-R., Shin S., Kim J.Y., Jung I.C., Lee M.Y. (2022). Anti-fatigue potential of *Pinus koraiensis* leaf extract in an acute exercise-treated mouse model. Biomed. Pharmacother..

[16] Choi Y., Yang C., Yoon J.S., Kim S., Kim S.-Y., Lee M.Y. (2025). Effect of *Pinus koraiensis* leaf extract on fatigue reduction and exercise performance: Study protocol for a randomized, double-blind, placebocontrolled clinical trial. Front. Med..

[17] Kwon K., Oh Y., Kim C., Yu C., Lee J. (2021). Biological activities and anti-wrinkle effects of *Pinus koraiensis* Siebold et Zucc. leaf extract. Korean J. Med. Crop Sci..

[18] Jo J.-B., Park H.-J., Lee E.-H., Lee J.-E., Lim S.-B., Hong S.-H., Cho Y.-J. (2017). Whitening and anti-wrinkle effect of *Pinus koraiensis* leaves extracts according to the drying technique. J. Appl. Biol. Chem..

[19] Hwang H.J., Yu J.-S., Lee H.Y., Kwon D.-J., Han W., Heo S.-I., Kim S.Y. (2014). Evaluations on deodorization effect and anti-oral microbial activity of essential oil from *Pinus koraiensis*. Korean J. Plant Resour..

[20] Kim J.-E., Han H., Xu Y., Lee M.-H., Lee H.-J. (2023). Efficacy of FRO on acne vulgaris pathogenesis. Pharmaceutics.

[21] Han H., Lee S.-O., Xu Y., Kim J.-E., Lee H.-J. (2022). SPHK/HIF-1α signaling pathway has a critical role in chrysin-induced anticancer activity in hypoxia-induced PC-3 cells. Cells.

[22] Han H., Kim J.-E., Lee H.-J. (2024). Effect of apigetrin in pseudo-SARS-CoV-2-induced inflammatory and pulmonary fibrosis in vitro model. Sci. Rep..

[23] Zhao X., Drlica K. (2014). Reactive oxygen species and the bacterial response to lethal stress. Curr. Opin. Microbiol..

[24] Hsu H.-Y., Wen M.-H. (2002). Lipopolysaccharide-mediated reactive oxygen species and signal transduction in the regulation of interleukin-1 gene expression. J. Biol. Chem..

[25] Simon F., Fernández R. (2009). Early lipopolysaccharide-induced reactive oxygen species production evokes necrotic cell death in human umbilical vein endothelial cells. J. Hypertens..

[26] Li Y., Ghasemi Naghdi F., Garg S., Adarme-Vega T.C., Thurecht K.J., Ghafor W.A., Tannock S., Schenk P.M. (2014). A comparative study: The impact of different lipid extraction methods on current microalgal lipid research. Microb. Cell Factories.

[27] Saini R., Guleria S., Kaul V.K., Lal B., Babu G.D.K., Singh B. (2010). Comparison of the volatile constituents of *Elsholtzia fruiticosa* extracted by hydrodistillation, supercritical fluid extraction and head space analysis. Nat. Prod. Commun..

[28] Costa P., Grosso C., Gonçalves S., Andrade P.B., Valentão P., Bernardo-Gil M.G., Romano A. (2012). Supercritical fluid extraction and hydrodistillation for the recovery of bioactive compounds from Lavandula viridis L’Hér. Food Chem..

[29] Masuda M., Era M., Kawahara T., Kanyama T., Morita H. (2015). Antibacterial effect of fatty acid salts on oral bacteria. Biocontrol Sci..

[30] Abdel-Aziz M.M., Emam T.M., Raafat M.M. (2020). Hindering of cariogenic *Streptococcus mutans* biofilm by fatty acid array derived from an endophytic *Arthrographis kalrae* strain. Biomolecules.

[31] Sengupta A., Ghosh M. (2012). Comparison of native and capric acid-enriched mustard oil effects on oxidative stress and antioxidant protection in rats. Br. J. Nutr..

[32] Lee S.I., Kang K.S. (2017). Function of capric acid in cyclophosphamide-induced intestinal inflammation, oxidative stress, and barrier function in pigs. Sci. Rep..

[33] Ameena M., Arumugham M., Ramalingam K., Shanmugam R. (2024). Biomedical applications of lauric acid: A narrative review. Cureus.

[34] Assiri M.A., Ali A., Ibrahim M., Khan M.U., Ahmed K., Akash M.S.H., Abbas M.A., Javed A., Suleman M., Khalid M. (2023). Potential anticancer and antioxidant lauric acid-based hydrazone synthesis and computational study toward the electronic properties. RSC Adv..

[35] Ramya V., Shyam K.P., Kowsalya E., Balavigneswaran C.K., Kadalmani B. (2022). Dual roles of coconut oil and its major component lauric acid on redox nexus: Focus on cytoprotection and cancer cell death. Front. Neurosci..

[36] Wei C.-C., Yen P.-L., Chang S.-T., Cheng P.-L., Lo Y.-C., Liao V.H.-C. (2016). Antioxidative activities of both oleic acid and *Camellia tenuifolia* seed oil are regulated by the transcription factor DAF-16/FOXO in *Caenorhabditis elegans*. PLoS ONE.

[37] Bhattacharjee B., Pal P.K., Chattopadhyay A., Bandyopadhyay D. (2020). Oleic acid protects against cadmium induced cardiac and hepatic tissue injury in male Wistar rats: A mechanistic study. Life Sci..

[38] Fratianni F., d’Acierno A., Ombra M.N., Amato G., De Feo V., Ayala-Zavala J.F., Coppola R., Nazzaro F. (2021). Fatty acid composition, antioxidant, and in vitro anti-inflammatory activity of five cold-pressed prunus seed oils, and their anti-biofilm effect against pathogenic bacteria. Front. Nutr..

[39] Santamarina A.B., Pisani L.P., Baker E.J., Marat A.D., Valenzuela C.A., Miles E.A., Calder P.C. (2021). Anti-inflammatory effects of oleic acid and the anthocyanin keracyanin alone and in combination: Effects on monocyte and macrophage responses and the NF-κB pathway. Food Funct..

[40] Mustafa A., Indiran M.A., Shanmugham R., Ramalingam K. (2023). Anti-inflammatory activity of lauric acid, thiocolchicoside and thiocolchicoside-lauric acid formulation. Bioinformation.

[41] Khan H.U., Aamir K., Jusuf P.R., Sethi G., Sisinthy S.P., Ghildyal R., Arya A. (2021). Lauric acid ameliorates lipopolysaccharide (LPS)-induced liver inflammation by mediating TLR4/MyD88 pathway in Sprague Dawley (SD) rats. Life Sci..

[42] Tang Y., Shen Y., Lai W., Yao C., Sui C., Hao T., Du J., Li Y., Mai K., Ai Q. (2025). Lauric acid ameliorates excessive linoleic acid induced macrophage inflammatory response and oxidative stress in large yellow croaker (*Larimichthys crocea*). Biochim. Et Biophys. Acta (BBA)-Mol. Cell Biol. Lipids.

[43] Pauls S.D., Rodway L.A., Winter T., Taylor C.G., Zahradka P., Aukema H.M. (2018). Anti-inflammatory effects of α-linolenic acid in M1-like macrophages are associated with enhanced production of oxylipins from α-linolenic and linoleic acid. J. Nutr. Biochem..

[44] Su H., Liu R., Chang M., Huang J., Wang X. (2017). Dietary linoleic acid intake and blood inflammatory markers: A systematic review and meta-analysis of randomized controlled trials. Food Funct..

[45] Miyamoto J., Mizukure T., Park S.-B., Kishino S., Kimura I., Hirano K., Bergamo P., Rossi M., Suzuki T., Arita M. (2015). A gut microbial metabolite of linoleic acid, 10-hydroxy-cis-12-octadecenoic acid, ameliorates intestinal epithelial barrier impairment partially via GPR40-MEK-ERK pathway. J. Biol. Chem..

[46] Kim N.-H., Park J.H., Koo D.-H., Lee T., Yang J.-Y., Lee H.-Y. (2022). Potential anticancer effect of sodium caprate on human gastric cancer cells. Hum. Exp. Toxicol..

[47] Narayanan A., Ananda Baskaran S., Amalaradjou M.A.R., Venkitanarayanan K. (2015). Anticarcinogenic properties of medium chain fatty acids on human colorectal, skin and breast cancer cells in vitro. Int. J. Mol. Sci..

[48] Mori S., Fujiwara-Tani R., Ogata R., Ohmori H., Fujii K., Luo Y., Sasaki T., Nishiguchi Y., Bhawal U.K., Kishi S. (2025). Anti-Cancer and Pro-Immune Effects of Lauric Acid on Colorectal Cancer Cells. Int. J. Mol. Sci..

[49] Lappano R., Sebastiani A., Cirillo F., Rigiracciolo D.C., Galli G.R., Curcio R., Malaguarnera R., Belfiore A., Cappello A.R., Maggiolini M. (2017). The lauric acid-activated signaling prompts apoptosis in cancer cells. Cell Death Discov..

[50] Verma P., Ghosh A., Ray M., Sarkar S. (2020). Lauric acid modulates cancer-associated microRNA expression and inhibits the growth of the cancer cell. Anti-Cancer Agents Med. Chem..

[51] Carrillo Pérez C., Cavia Camarero M.D.M., Alonso de la Torre S. (2012). Antitumor effect of oleic acid; mechanisms of action. A review. Nutr. Hosp..

[52] Giulitti F., Petrungaro S., Mandatori S., Tomaipitinca L., De Franchis V., D’Amore A., Filippini A., Gaudio E., Ziparo E., Giampietri C. (2021). Anti-tumor effect of oleic acid in hepatocellular carcinoma cell lines via autophagy reduction. Front. Cell Dev. Biol..

[53] Kim J.S., Kim D.K., Moon J.Y., Lee M.-Y., Cho S.K. (2024). Oleic acid inhibits the migration and invasion of breast cancer cells with stemness characteristics through oxidative stress-mediated attenuation of the FAK/AKT/NF-κB pathway. J. Funct. Foods.

[54] Qiu J., Zhao Z., Suo H., Paraghamian S.E., Hawkins G.M., Sun W., Zhang X., Hao T., Deng B., Shen X. (2024). Linoleic acid exhibits anti-proliferative and anti-invasive activities in endometrial cancer cells and a transgenic model of endometrial cancer. Cancer Biol. Ther..

[55] Domagała D., Leszczyńska T., Koronowicz A., Domagała B., Drozdowska M., Piasna-Słupecka E. (2021). Mechanisms of Anticancer Activity of a fatty acid mixture extracted from Hen Egg Yolks Enriched in Conjugated Linoleic Acid Diene (CLA) against WM793 melanoma cells. Nutrients.

[56] González A., Fullaondo A., Rodríguez J., Tirnauca C., Odriozola I., Odriozola A. (2025). Conjugated linoleic acid metabolite impact in colorectal cancer: A potential microbiome-based precision nutrition approach. Nutr. Rev..

[57] Bergsson G., Arnfinnsson J., Steingrímsson O., Thormar H. (2001). In vitro killing of *Candida albicans* by fatty acids and monoglycerides. Antimicrob. Agents Chemother..

[58] Jadhav A., Mortale S., Halbandge S., Jangid P., Patil R., Gade W., Kharat K., Karuppayil S.M. (2017). The dietary food components capric acid and caprylic acid inhibit virulence factors in *Candida albicans* through multitargeting. J. Med. Food.

[59] Kim J.-H., Kim Y.-H., Park B.-I., Choi N.-Y., Kim K.-J. (2023). *Pinus koraiensis* essential oil attenuates the pathogenicity of superbacteria by suppressing virulence gene expression. Molecules.

[60] Bakkali F., Averbeck S., Averbeck D., Idaomar M. (2008). Biological effects of essential oils—A review. Food Chem. Toxicol..

[61] Zahraoui E.M. (2025). Essential Oils: Antifungal activity and study methods. Moroc. J. Agric. Sci..

[62] Contreras-Martínez O.I., Angulo-Ortíz A., Santafé Patiño G., Sierra Martinez J., Berrio Soto R., de Almeida Rodolpho J.M., de Godoy K.F., de Freitas Aníbal F., de Lima Fragelli B.D. (2024). Synergistic antifungal effect and In vivo toxicity of a monoterpene Isoespintanol obtained from *Oxandra xylopioides* Diels. Molecules.

[63] Kalemba D., Kunicka A. (2003). Antibacterial and antifungal properties of essential oils. Curr. Med. Chem..

[64] Ashour M., Wink M., Gershenzon J. (2010). Biochemistry of terpenoids: Monoterpenes, sesquiterpenes and diterpenes. Annual Plant Reviews Volume 40: Biochemistry of Plant Secondary Metabolism.

[65] Carvalho L.A., Queijo R.G., Baccaro A.L., Siena A.D., Silva W.A., Rodrigues T., Maria-Engler S.S. (2022). Redox-related proteins in melanoma progression. Antioxidants.

[66] Neittaanmäki N., Zaar O., Cehajic K.S., Nilsson K.D., Katsarelias D., Bagge R.O., Paoli J., Fletcher J.S. (2024). ToF-SIMS imaging reveals changes in tumor cell lipids during metastatic progression of melanoma. Pigment Cell Melanoma Res..

[67] Lumaquin-Yin D., Montal E., Johns E., Baggiolini A., Huang T.-H., Ma Y., LaPlante C., Suresh S., Studer L., White R.M. (2023). Lipid droplets are a metabolic vulnerability in melanoma. Nat. Commun..

[68] Łuczaj W., Dobrzyńska I., Skrzydlewska E. (2023). Differences in the phospholipid profile of melanocytes and melanoma cells irradiated with UVA and treated with cannabigerol and cannabidiol. Sci. Rep..

[69] Venn-Watson S., Schork N.J. (2023). Pentadecanoic acid (C15: 0), an essential fatty acid, shares clinically relevant cell-based activities with leading longevity-enhancing compounds. Nutrients.

[70] To N.B., Nguyen Y.T.-K., Moon J.Y., Ediriweera M.K., Cho S.K. (2020). Pentadecanoic acid, an odd-chain fatty acid, suppresses the stemness of MCF-7/SC human breast cancer stem-like cells through JAK2/STAT3 signaling. Nutrients.

